# A CONSORT‐guided, randomized controlled clinical trial of nebulized administration of dexamethasone and saline on lower airway cytokine mRNA expression in horses with moderate asthma

**DOI:** 10.1111/jvim.16983

**Published:** 2024-01-11

**Authors:** Stephanie Bond, Renaud Léguillette

**Affiliations:** ^1^ Faculty of Veterinary Medicine University of Calgary Calgary Alberta Canada; ^2^ School of Veterinary Science, Faculty of Science University of Queensland Gatton Queensland Australia

**Keywords:** cytokine expression, mild equine asthma, mRNA, one health, qPCR, respirable dust, REST analysis

## Abstract

**Background:**

Nebulized administration of dexamethasone on cytokine regulation in horses with moderate asthma has not been investigated.

**Objective:**

To investigate the changes in expression of inflammatory cytokine mRNA after nebulized administration of dexamethasone treatment of horses with moderate asthma.

**Animals:**

Horses with naturally occurring moderate asthma (n = 16) and healthy control horses (n = 4). All horses were kept in a dusty environment during the study.

**Methods:**

Prospective, parallel, randomized, controlled, blinded clinical trial. Blood endogenous cortisol, tracheal mucus, and bronchoalveolar lavage (BAL) were sampled before and after 13 days treatment with either nebulized administration of dexamethasone (15 mg once daily) or 0.9% saline (3 mL). Treatment groups were randomly allocated via randomization function (Microsoft Excel). Amplification of target mRNA in BAL fluid (IL‐1β, IL‐4, IL‐5, IL‐6, IL‐8, IL‐10, IL‐12, IL‐17, IL‐23, IFN‐γ, Eotaxin‐2, and TNF‐α) was achieved by qPCR, and the relative expression software tool was used to analyze BAL inflammatory cytokine mRNA.

**Results:**

Horses treated with nebulized administration of dexamethasone had increased relative expression of IL‐5 (1.70‐fold), IL‐6 (1.71‐fold), IL‐17 (3.25‐fold), IL‐12 (1.66‐fold), and TNF‐α (1.94‐fold), and decreased relative expression of IL‐23 (1.76‐fold; *P* = .04) in samples collected on Day 14, in comparison to samples collected on Day 0 (all *P* < .05). Horses treated with nebulized administration of saline had no significant difference in the relative expression of any gene (all *P* > .05).

**Conclusions and Clinical Importance:**

Nebulized administration of dexamethasone was associated with increased expression of inflammatory cytokine mRNA. There was no improvement in inflammatory airway cytology associated with either dexamethasone or saline treatment.

**Abbreviations**
BAL: bronchoalveolar lavage
GAPDH: glyceraldehyde‐3P‐dehydrogenase
HPRT: hypoxanthine ribosyltransferase
RPL32: ribosomal protein L32
SDHA: succinate dehydrogenase complex subunit A

## 1 INTRODUCTION

Nebulization of injectable dexamethasone does not induce airway inflammation and has minimal systemic bioavailability in the healthy horse.^1^ However, nebulized administration of dexamethasone (5 mg q24h) does not improve lung mechanics in horses with severe asthma, whereas significantly suppressing endogenous cortisol production.^2^, ^3^ Recruitment of inflammatory cells is driven through increased production of inflammatory mediators, associated with increased activity of transcription factors. The efficacy of systemic corticosteroids on inflammatory gene expression in both bronchoalveolar lavage derived cells^4^, ^5^, ^6^ and bronchial epithelium^5^ has therefore been evaluated in horses with severe asthma. Systemic dexamethasone treatment (at 20 mg once daily) in horses with mild asthma is associated with lower levels of IL‐5 mRNA, inhibiting a dysregulated T2 response in bronchoalveolar lavage derived cells after allergen exposure.^7^ Excessive tracheal mucus accumulation is a feature of moderate asthma^8^ and increased mucociliary clearance would also be of benefit. Nebulized administration of saline is often used as a placebo in treatment trials investigating human patients with chronic obstructive pulmonary disease, as it relieves breathlessness—potentially by facilitating an increase in mucociliary clearance—without affecting lung function.^9^

Corticosteroid treatment aims to reduce clinical signs of asthma through reducing inflammation. Based on the high percentage of respirable size droplets produced by equine nebulizers and the high lung bioavailability of antibiotics administered by nebulization in horses,^10^, ^11^ it has been assumed that nebulized administration of dexamethasone has good bioavailability in the lower airways of horses,^1^ however, this has not been verified. Given the local route of administration usually requires lower dosages, but that 5 mg lacked clinical effectiveness in a more severe phenotype of asthma, it is unknown whether a higher dose of dexamethasone sodium phosphate (15 mg) administered via nebulization would be effective at treating horses with moderate asthma. Although limiting respirable particulate matter exposure is recognized as the most effective way of improving clinical signs and airway inflammation associated with asthma in horses, this is often challenging for clients to accomplish. The development of an exaggerated lower airway inflammatory response is potentially because of interactions between environmental factors and immunological or genetic predisposition. However, exposure to an antigenic environment elicits an immune response in the respiratory tract of healthy horses and humans; this healthy, normal response appears to be dose‐dependent.^12^, ^13^, ^14^, ^15^

The objectives of this study were therefore to: (a) assess the impact of maintaining healthy horses in a dusty environment on inflammatory cytokine expression in bronchoalveolar lavage derived cells, and (b) investigate the effects of nebulized administration of dexamethasone or saline. Our hypotheses were: that in an environment with high levels of exposure to respirable particulate matter (a) nebulized administration of dexamethasone, but not saline, would suppress an inflammatory response in horses with chronic airway inflammation, and (b) the antigenic environment would elicit an immune response in the respiratory tract of healthy horses.

## 2 MATERIALS AND METHODS

### 2.1 Animals and study design

This study was conducted in accordance with the recommendations of the Canadian Council of Animal Care. The research protocol was reviewed and approved by the University of Calgary Veterinary Sciences Animal Care Committee (AC17‐0097). This was a prospective, randomized, controlled, blinded clinical trial performed adhering to CONSORT guidelines,^16^ with the participation of the UCVM class of 2019. Gelding horses (n = 20, 435‐612 kg) were enrolled from 2 large client‐owned herds that, before the study, resided on 2 properties (Lake Louise and Cochrane, AB, Canada). Horses either had a chronic history of nasal mucus and coughing (moderate asthma; n = 16), or no history of respiratory disease (healthy controls; n = 4). Horses had been under the routine care of an external veterinarian for several years and were assessed by this veterinarian before transport. Inclusion criteria during this screening stage were a clinical examination within normal limits, aside from the observation of nasal mucus and coughing, and no evidence of systemic illness on complete blood count or biochemistry analysis. Horses were transferred to a dusty indoor stable a week before initial sampling to enable habituation to the environment. Individual stalls which permitted free movement of air between stalls with straw bedding were used. Horses were fed grass hay suspended in hay nets for the duration of the trial and were given free access to water. Horses were lightly exercised every second day (10 minutes trot on a lunge line), and cough counting was performed during exercise (Table 1) and at rest during stall cleaning (coughs were counted over a period of 30 to 45 minutes) by observers blinded to the horses' treatment groups. Dust concentrations of particulates <4 μm (g/m^3^) were measured at a central location in the stable, 1.8 m above ground level for 4‐minute sampling periods every 4 hours throughout the trial (SidePak AM520, TSI, Shoreview, Minnesota). Dust concentration data were analyzed using commercially available software (TrakPro 5, TSI, Shoreview, Minnesota).

**TABLE 1:** Median (±IQR) cough rates (per minute) at rest (30‐45 minutes during stall cleaning) and during lunging exercise (10 minutes trot, every second day) after daily nebulization with 15 mg dexamethasone sodium phosphate (n = 8), 3 mL 0.9% saline (n = 8), or no treatment in environmental control horses (n = 4).

	Dexamethasone		Saline		Control	
	Rest	Exercise	Rest	Exercise	Rest	Exercise
Days −7‐0	0.05 (0‐0.25)	0.75 (0‐2.50)	0.02 (0‐0.05)	0.25 (0‐0.88)	0.09 (0‐0.08)	0.25 (0‐3.00)
Days 1‐7	0.07 (0‐0.18)	0.57 (0.43‐0.57)	0.05 (0.03‐0.06)	1.00 (0.63‐1.13)	0.22 (0‐0.30)	1.25 (0.75‐1.50)
Days 8‐14	0.08 (0‐0.19)	0.71 (0.57‐1.86)	0.08 (0‐0.20)	0.88 (0.50‐1.17)	0.25 (0.06‐1.50)	1.00 (1.00‐1.25)

### 2.2 Treatment groups

Horses were weighed, and sampling procedures (see below) were performed on all horses (n = 20) on Day 0 and Day 14. On Day 1, horses were allocated into 1 of 3 treatment groups (*Dex, Saline, Control*) based on their disease status (see below for inclusion criteria; moderate asthma vs healthy) and random treatment group selection (horses with moderate asthma), using the randomization function of a commercially available software program (Excel, Microsoft 365 apps for enterprise, Calgary, Alberta). Horses were enrolled in the healthy *Control* group based on a clinical examination within normal limits, no history of respiratory disease, coughing, or nasal mucus, an endoscopic tracheal mucus score of 0,^17^ and a noninflammatory bronchoalveolar lavage fluid profile (mast cell ≤ 2%; eosinophils ≤ 0.5%; neutrophils ≤ 5%). Horses were considered to have moderate asthma based on the following inclusion criteria: (a) inflammatory bronchoalveolar lavage with an increased percentage of mast cells (>2%) or/and eosinophils (>0.5%) or/and neutrophils (>5%); (b) History of nasal mucus, coughing or both, confirmed during clinical examination; (c) absence of labored breathing at rest.^8^ The *Dex* group was treated with 15 mg (5 mg/mL; 3 mL total volume) dexamethasone sodium phosphate (Dexamethasone 5, Vetoquinol, Lavaltrie, Quebec) once daily via nebulization (horses with moderate asthma; n = 8; Flexineb E3, Nortev, Oranmore, Ireland). The *Saline* group was treated with 3 mL of 0.9% saline administered via nebulization once daily (horses with moderate asthma; n = 8). The *Control* group was a no treatment environmental control (healthy horses; n = 4). All horses in the *Dex* and *Saline* groups were treated for 13 days, and the sampling procedures were repeated on Day 14. No other medications were given to horses for the duration of the trial. Those administering treatments were blinded to the treatment provided to the *Dex* and *Saline* groups, as were the specialists who reported the bronchoalveolar lavage results and those dealing with laboratory investigations.

### 2.3 Sampling procedures

Before horses were moved out of their stalls, blood was sampled from the jugular vein between 7 and 8 am on Day 0, 7, and 14, serum was separated and immediately frozen for endogenous cortisol quantification. Horses were then pre‐medicated with acepromazine maleate (0.07‐0.08 mg/kg, IM or IV), and sedated with xylazine hydrochloride (0.4‐0.5 mg/kg, IV) and butorphanol tartrate (0.015‐0.02 mg/kg IV).

Endoscopic scoring of airway mucus quantity was performed as previously validated^17^ using an average score between 5 observers (RL, SB and 3 students from the UCVM Class of 2019).

A bronchoalveolar lavage was performed as previously described.^18^ Briefly, a balloon‐tipped bronchoalveolar lavage tube (SKU: BAL300, Mila International, Florence, Kentucky) was inserted until wedged against the wall of a bronchus, and 2 boluses (250 mL/bolus) of sterile isotonic saline (0.9% NaCl) solution were sequentially instilled. Lavage fluid was recovered, and 2 10 mL aliquots were immediately stored at 4°C for cytological analysis. A differential count of bronchoalveolar lavage fluid was performed on a minimum of 400 cells for allocation of treatment groups.^19^ Preparation of slides was performed with 400 μL of bronchoalveolar lavage fluid within 4 hours of sample collection, which was centrifuged using a Cytospin (90 × *g* for 5 minutes) and stained with modified Wright‐Giemsa stain. Two 50 mL aliquots of bronchoalveolar lavage fluid were centrifuged at 700 × *g* for 10 minutes immediately after collection; the supernatant was then discarded, and the cell pellets resuspended in 1.5 mL of RNAprotect (Qiagen, Mississauga, Ontario, Canada). Samples were stored at −80°C until extraction.

### 2.4 RNA extraction, cDNA synthesis, and qPCR analysis

Total RNA was extracted from bronchoalveolar lavage samples using the RNeasy Mini Kit (Qiagen, Mississauga, Ontario, Canada), as per manufacturer instructions, using 40 μL RNase‐free water to elute samples. Contaminating genomic DNA was removed before cDNA synthesis using dsDNase (Thermo Fisher Scientific, #EN0771, Wilmington, Delaware). Approximately 500 ng total RNA was retro‐transcribed with the Omniscript Reverse Transcription Kit (Qiagen, Mississauga, Ontario), as per manufacturer instructions, with RNaseOUT (Thermo Scientific, Wilmington, Delaware) and Oligo(dT) primers (Invitrogen, Burlington, Ontario, Canada) included in the reaction mixture. Amplification of target RNA (IL‐1β, IL‐4, IL‐5, IL‐6, IL‐8 (CXCL‐8), IL‐10, IL‐12, IL‐17, IL‐23, IFN‐γ, Eotaxin‐2, and TNF‐α) was performed using previously optimized reaction conditions,^20^, ^21^, ^22^ and primer sequences.^7^ Reference genes included glyceraldehyde‐3P‐dehydrogenase (GAPDH), succinate dehydrogenase complex subunit A (SDHA), hypoxanthine ribosyltransferase (HPRT), and ribosomal protein L32 (RPL32), which have been shown to provide accurate normalization for gene expression studies in bronchoalveolar lavage fluid from horses with moderate asthma, treated with dexamethasone.^23^ Reactions were executed in triplicate, with template from samples collected on Day 0 and Day 14 from the same horse included on the same plate. Negative and No RT controls were included on each plate. Quantification cycle (Cq) results <45 were classified as positive.

### 2.5 Statistical analysis

The relative expression software tool (REST), which allows for correction for PCR efficiency and normalization with multiple reference genes,^24^ was used to compare cytokine gene expression in samples.^20^ Calculations were performed using a reference gene expression normalization factor based on a geometric mean of 4 housekeeping genes previously validated for BAL analysis in horses with moderate asthma.^23^ If an inflammatory gene was not present in detectable quantities at either timepoint in a particular treatment group it was excluded from analysis.

Normality of the distribution of the BALF differential cell counts, serum cortisol, and averaged tracheal mucus scores were confirmed by a Shapiro‐Wilk normality test. A 2‐way repeated measures ANOVA (controlling for treatment group and timepoint [Day 0 vs Day 14]) was used to assess differences between groups, except for mucus scores (ordinal data) that were assessed using a Kruskal‐Wallis test. A zero‐inflated poisson mixed effects model was used to assess differences in the cough counts between groups during the study. A *P*‐value <.05 was considered significant. Data were shown as mean ± SD when normally distributed. Tracheal mucus scoring data were presented as median (range).

## 3 RESULTS

### 3.1 Clinical signs

One horse had a heel bulb wound after transportation to the study site that was treated with topical therapy and bandaging and resolved without complications. Another horse had a fractured left frontal bone after a head trauma in the stall 2 days after arrival during the habituation period and received 2.2 mg/kg of phenylbutazone for 3 days and topical therapy; it healed well with no complications. No horse developed labored breathing or tachypnea at rest at any time during the study, and the only respiratory clinical signs noted were intermittent nasal discharge (mostly after exercise or in the morning) and cough at rest and during the lunging exercise. There was no significant group or time effect (*P* = .15) on the cough scores at rest nor during the exercise sessions (Table 1).

### 3.2 Dust

There was a steady circadian pattern associated with animal husbandry (ie, stall cleaning, feeding, lunging) between 0.05 and 0.1 mg/m^3^. Dust concentration data has been previously reported.^25^

### 3.3 Tracheal mucus

There was no significant difference in tracheal mucus score in any group (*Dex*, *Saline*, *Control*) at Day 0 or Day 14 and over time (Day 0 vs Day 14). Tracheal mucus scores of the *Dex* group were 2.7 (0.6‐4.3) on Day 0 and 2.4 (1.6‐3.3) on Day 14. Tracheal mucus scores of the *Saline* group were 1.2 (0‐4.4) on Day 0 and 2.2 (0.9‐4.4) on Day 14. Tracheal mucus scores of the *Control* group were 0.9 (0‐2.2) on Day 0 and 3.0 (1.0‐4.2) on Day 14.

### 3.4 Cytology

**FIGURE 1:**
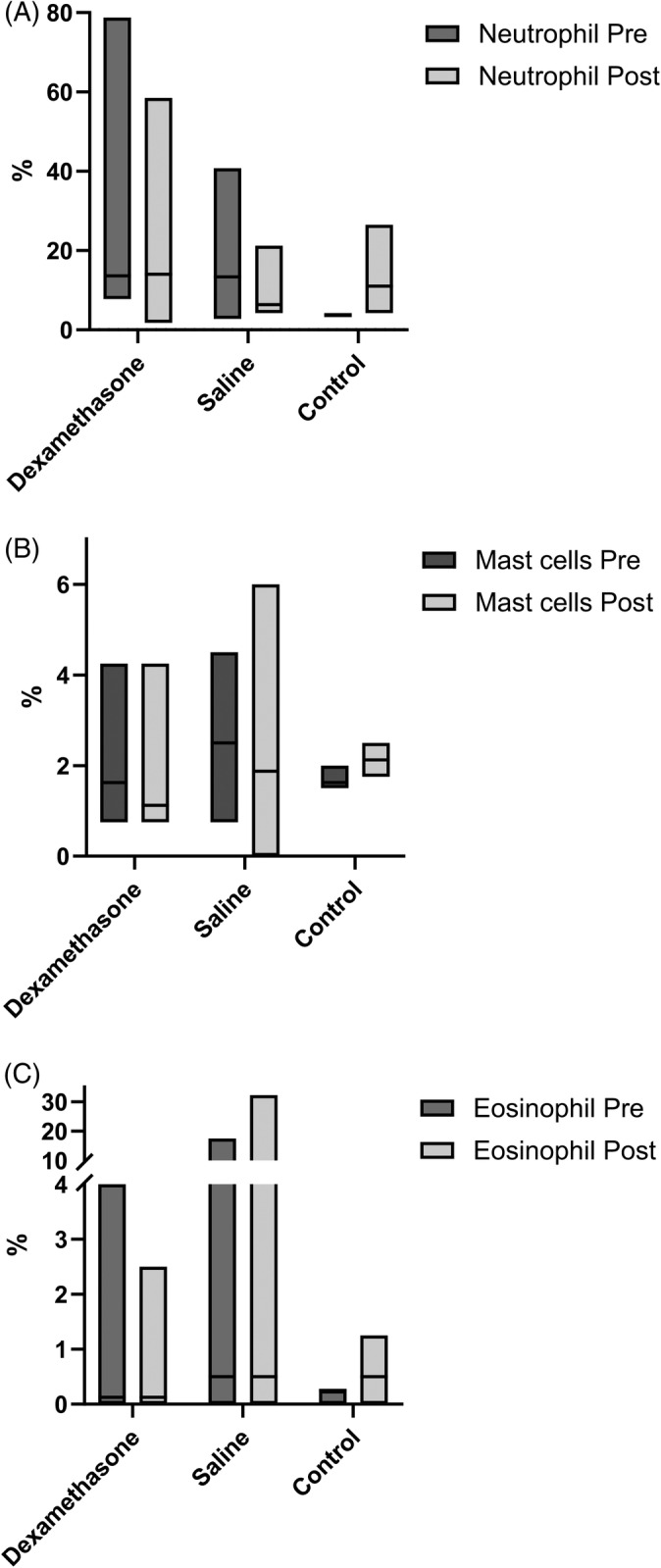
Bronchoalveolar lavage fluid differential cell counts in 16 horses with moderate asthma before (Pre: Day 0) and after (Post: Day 14) treatment with nebulized dexamethasone (15 mg q24h, n = 8), or 0.9% nebulized saline (3 mL q24h, n = 8). Bronchoalveolar lavage fluid differential cell counts for no treatment controls (n = 4) are also provided. (A) Differential neutrophils' counts. (B) Differential mast cells' count. (C) Differential eosinophils' count.

Individual bronchoalveolar lavage fluid differential cell counts for each horse on Day 0 and Day 14 are shown in Figure 1. There was no significant difference in the proportion of any cell type either between treatment groups on Day 0 (neutrophil, *P* = .38; eosinophil, *P* = .42; mast cell, *P* = .53; alveolar macrophage, *P* = .42; lymphocyte, *P* = .74), or between Day 0 and Day 14 (neutrophil, *P* = .58; eosinophil, *P* = .42; mast cell, *P* = .67; alveolar macrophage, *P* = .69; lymphocyte, *P* = .57). However, on Day 14, only 1 horse in the *Control* group still had bronchoalveolar lavage cytology within normal limits,^8^ whereas the other 3 horses had developed airway inflammation (n = 1, neutrophilic inflammation; n = 2, mixed inflammatory profile; Figure 1).

### 3.5 Cortisol

There was a significant decrease in blood endogenous cortisol in the *Dexamethasone* group between Day 0 (109 ± 39 nmol/L) and Days 7 (47 ± 21 nmol/L) and 14 (50 ± 16 nmol/L), and in the *Saline* group between Day 0 (111 ± 38 nmol/L) and Day 7 (87 ± 33 nmol/L) only.

### 3.6 Gene expression

**FIGURE 2:**
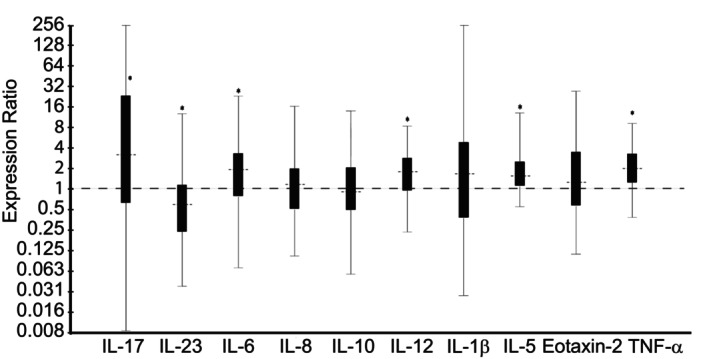
Effect of 13 days of treatment with nebulized dexamethasone (15 mg q24h; Day 14 vs Day 0) on relative expression of inflammatory cytokine mRNA from bronchoalveolar lavage‐derived cells obtained from 8 horses with moderate asthma. An expression ratio of 1 indicates that there was no change in the relative expression of that gene over time (>1 indicates up‐regulation; <1 indicates down‐regulation). Asterisk (*) indicates that the change in relative expression was significant (*P* < .05).

*Dexamethasone* treatment group; Day 14 vs Day 0: Horses treated with nebulized administration of dexamethasone had increased relative expression of IL‐5 (1.70‐fold; *P* = .001), IL‐6 (1.71‐fold; *P* = .04), IL‐17 (3.25‐fold; *P* = .033), IL‐12 (1.66‐fold; *P* = .007), and TNF‐α (1.94‐fold; *P* = .001), and decreased relative expression of IL‐23 (1.76‐fold; *P* = .04) in samples collected on Day 14, in comparison to samples collected on Day 0 (Figure 2). There was no significant change in relative expression levels of IL‐1β, IL‐8, IL‐10, or Eotaxin‐2 associated with nebulized administration of dexamethasone (Figure 2).

**FIGURE 3:**
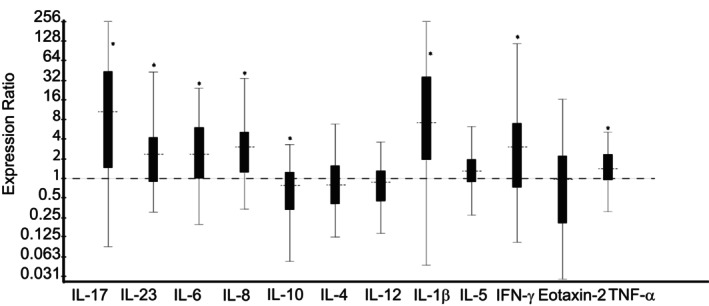
Relative expression of inflammatory cytokine mRNA from bronchoalveolar lavage‐derived cells obtained on Day 14, in horses with moderate asthma treated with nebulized dexamethasone (15 mg q24h), in comparison to no‐treatment control horses. An expression ratio of 1 indicates that there was no difference in the relative expression of that gene between groups (>1 indicates up‐regulation; <1 indicates down‐regulation). Asterisk (*) indicates that the difference in relative expression was significant (*P* < .05).

*Dexamethasone* treatment group vs no‐treatment *Control* group; Day 14: Horses treated with nebulized administration of dexamethasone had increased relative expression of IL‐1β (7.31‐fold; *P* < .001), IL‐6 (2.34‐fold; *P* = .003), IL‐8 (2.74‐fold; *P* < .001), IL‐17 (9.59‐fold; *P* < .001), IL‐23 (2.28‐fold; *P* = .001), IFN‐γ (3.03‐fold; *P* = .003), and TNF‐α (1.47‐fold; *P* = .007), and decreased relative expression of IL‐10 (1.63‐fold; *P* = .03) in comparison to no‐treatment *Control* horses on Day 14 (Figure 3). There was no significant difference in relative expression levels of IL‐4, IL‐5, IL‐12, or Eotaxin‐2 between groups (Figure 3).

**FIGURE 4:**
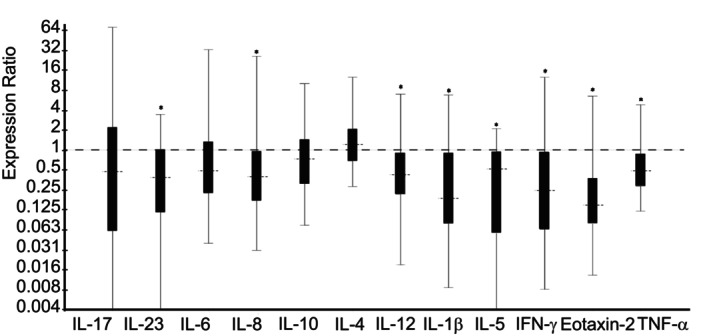
Relative expression of inflammatory cytokine mRNA from bronchoalveolar lavage‐derived cells obtained on Day 14, in horses with moderate asthma treated with nebulized 0.9% saline (3 mL q24h), vs treated with nebulized dexamethasone (15 mg q24h). An expression ratio of 1 indicates that there was no difference in the relative expression of that gene between groups (>1 indicates up‐regulation; <1 indicates down‐regulation). Asterisk (*) indicates that the difference in relative expression was significant (*P* < .05).

*Saline* treatment group vs *Dexamethasone* treatment group; Day 14: Horses treated with nebulized administration of saline had decreased relative expression of IL‐1β (3.91‐fold; *P* < .001), IL‐5 (5.38‐fold; *P* < .001), IL‐8 (2.13‐fold; *P* = .01), IL‐12 (2.38‐fold; *P* < .001), IL‐23 (6.45‐fold; *P* = .001), IFN‐γ (4.10‐fold; *P* = .002), Eotaxin‐2 (4.88‐fold; *P* < .001), and TNF‐α (1.87‐fold; *P* = .001), in comparison to horses treated with nebulized administration of dexamethasone on Day 14 (Figure 4). There was no significant change in relative expression levels of IL‐4, IL‐6, IL‐10, or IL‐17 (Figure 4).

**FIGURE 5:**
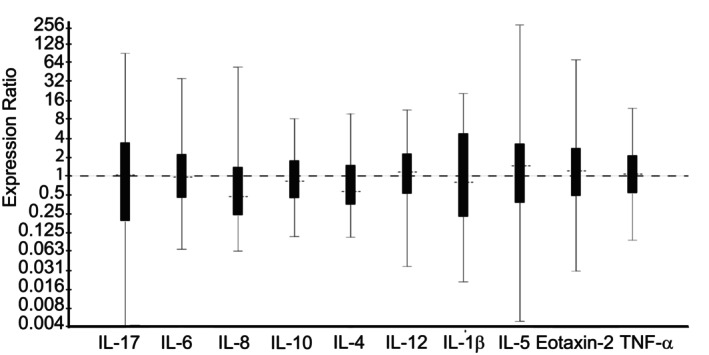
Effect of 13 days of treatment with nebulized 0.9% saline (3 mL, SID; Day 14 vs Day 0) on relative expression of inflammatory cytokine mRNA from bronchoalveolar lavage‐derived cells obtained from 8 horses with moderate asthma. An expression ratio of 1 indicates that there was no change in the relative expression of that gene over time (>1 indicates up‐regulation; <1 indicates down‐regulation). Asterisk (*) indicates that the change in relative expression was significant (*P* < .05).

*Saline* treatment group; Day 14 vs Day 0: Horses treated with nebulized administration of saline had no significant difference in the relative expression of any gene investigated (Day 14 vs Day 0; Figure 5).

**FIGURE 6:**
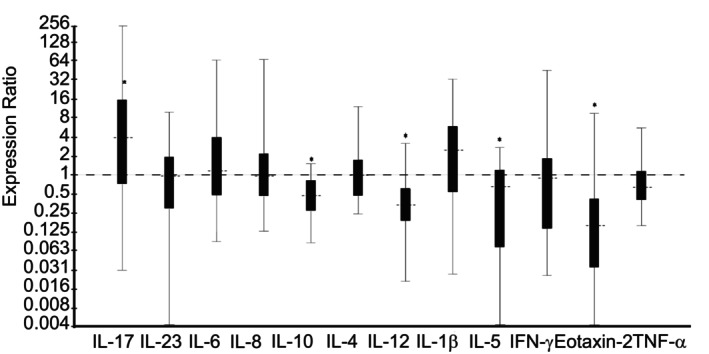
Relative expression of inflammatory cytokine mRNA from bronchoalveolar lavage‐derived cells obtained on Day 14, in horses with moderate asthma treated with nebulized 0.9% saline (3 mL q24h), in comparison to no‐treatment control horses. An expression ratio of 1 indicates that there was no difference in the relative expression of that gene between groups (>1 indicates up‐regulation; <1 indicates down‐regulation). Asterisk (*) indicates that the difference in relative expression was significant (*P* < .05).

*Saline* treatment group vs no‐treatment *Control* group; Day 14: Horses treated with nebulized administration of saline had decreased relative expression of IL‐5 (4.17‐fold; *P* = .003), IL‐10 (2.15‐fold; *P* < .001), IL‐12 (3.06‐fold; *P* < .001), and Eotaxin‐2 (7.19‐fold; *P* < .001), and increased relative expression of IL‐17 (3.85‐fold; *P* = .004), in comparison to control horses on Day 14 (Figure 6). There was no significant change in relative expression levels of IL‐1β, IL‐4, IL‐6, IL‐8, IL‐23, IFN‐γ, or TNF‐α (Figure 6).

**FIGURE 7:**
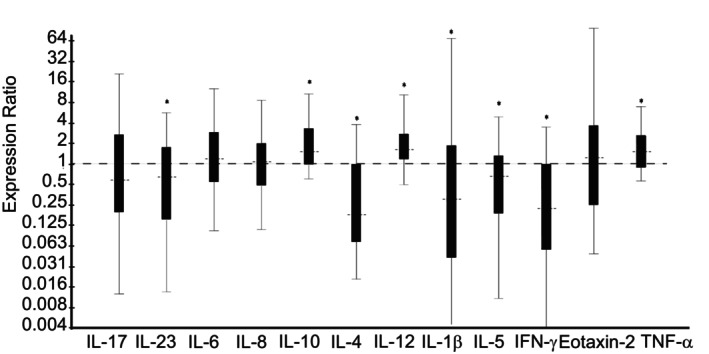
Effect of 13 days in a dusty environment (Day 14 vs Day 0) on relative expression of inflammatory cytokine mRNA from bronchoalveolar lavage‐derived cells obtained from no‐treatment control horses (n = 4). An expression ratio of 1 indicates that there was no change in the relative expression of that gene over time (>1 indicates up‐regulation; <1 indicates down‐regulation). Asterisk (*) indicates that the change in relative expression was significant (*P* < .05).

No‐treatment *Control* group; Day 14 vs Day 0: Prolonged exposure of *Control* horses to a dusty, stabled environment resulted in down‐regulation of IL‐23 (2.01‐fold; *P* = .03), IL‐4 (3.85‐fold; *P* < .001), IL‐5 (2.35‐fold; *P* = .008), IL‐1β (4.10‐fold; *P* = .01) and IFN‐γ (5.03‐fold; *P* < .001; n = 1 horse), and up‐regulation of IL‐10 (1.75‐fold; *P* = .001), IL‐12 (1.93‐fold; *P* < .001) and TNF‐α (1.54‐fold; *P* = .001) at Day 14, in comparison to samples collected at Day 0 (Figure 7). There was no significant change in relative expression levels of IL‐6, IL‐8, IL‐17, or Eotaxin‐2 in the bronchoalveolar lavage fluid from *Control* horses between Day 0 and Day 14 (Figure 7).

## 4 DISCUSSION

Nebulized administration of dexamethasone was associated with up‐regulation of inflammatory cytokines in horses with chronic airway inflammation, both in comparison to control horses, and asthmatic horses treated with nebulized administration of saline. In contrast, treatment of asthmatic horses with nebulized administration of saline was associated with down‐regulation of inflammatory cytokines, when compared to asthmatic horses treated with nebulized administration of dexamethasone, as well untreated healthy *Control* horses. There was no significant difference in the relative expression of any gene over time in horses treated with nebulized administration of saline. Prolonged exposure to high levels of respirable particulate matter was associated with the development of cytologic airway inflammation in healthy horses associated with a mixed inflammatory cytokine response.

The 15 mg daily dosage of dexamethasone administered via nebulization used in the present study was greater than described in 2 trials of horses with severe asthma trials.^2^, ^3^ The reason for this was the lack of improvement in lung mechanics reported with a dosage of 5 mg dexamethasone sodium phosphate nebulized once daily in horses with severe asthma in both previous studies.^2^, ^3^ We hypothesized that a higher dose of nebulized administration of dexamethasone sodium phosphate (15 mg) would be effective at treating horses with a less severe phenotype of asthma; we also used the same commercial dexamethasone sodium phosphate product as previous studies in severe asthma.^4^, ^5^ Furthermore, local administration typically requires a lower treatment dose than systemic treatment; dexamethasone treatment at a rate of 20 mg once daily is associated with decreased levels of IL‐5 mRNA, inhibiting a dysregulated T2 response in bronchoalveolar lavage derived cells from horses with mild asthma after allergen exposure.^7^ Since we expected that the effect of environmental conditions would sustain lung inflammation, we used a dosage of dexamethasone that would maximize its anti‐inflammatory effects, yet be a lower treatment dosage than has been systemically reported.^7^ Studies in horses with severe asthma report a rapid significant decrease in serum cortisol concentration after 3 (reduction: 52.4 nmol/L [converted from 1.9 μg/dL])^2^ and 7 (reduction: 57.9 nmol/L [converted from 2.1 μg/dL]^2^; 44.7 nmol/L^3^) days of nebulized administration of dexamethasone (5 mg q24h). Consistent with these studies,^2^, ^3^ we observed a significant decrease in blood endogenous cortisol at Day 7 and Day 14 of 62 and 59 nmol/L respectively, and as expected, the magnitude of cortisol suppression was greater than that observed at the lower dose rate (5 mg). Unlike in the healthy horse where the nebulization of injectable dexamethasone has minimal systemic bioavailability,^1^ the present study confirmed that systemic absorption occurs in horses with asthma, either though deposition in the upper airways, through swallowing, or via the lungs. The first studies investigating the effects of nebulized administration of dexamethasone in horses were aimed at testing its safety, and used a 5 mg dosage based on the fact that 1 mg dexamethasone was reported to be effective in humans with asthma, and that horses are expected to have a 5 times greater lung alveoli surface than humans.^1^ At 5 mg, dexamethasone sodium phosphate does not induce lung inflammation (detected using bronchoalveolar lavage cytology) and decreases lung neutrophilia in healthy horses that are stabled.^1^ Similarly, we did not observe changes in the BAL fluid cytology between the saline and dexamethasone groups. However, nebulizing 15 mg of sodium phosphate dexamethasone was associated with up‐regulation of IL‐1β, IL‐6, IL‐8, IL‐17, IL‐23, IFN‐γ, and TNF‐α, and down‐regulation of IL‐10, in comparison to control horses. Upregulation of TNF‐α plays an important role in the development of airway inflammation by enhancing T2 immune responses related to asthma pathogenesis. IL‐23 is an IL‐12‐related cytokine essential for the maintenance of T17 cells,^26^ and plays a key role in the development of inflammatory disease. Typically, inflammatory cytokine production is inhibited by glucocorticoid administration, and it is possible that the dose rate used in this study (15 mg SID) might have induced the immune response observed because of the preservatives in the dexamethasone sodium phosphate. Indeed, a previous study reported coughing during administration of this formulation in horses with severe asthma during administration, and hypothesized that it could be because of the vehicle.^3^ Alternatively, activation of the T1, T2, and T17 pathways was potentially because of prolonged exposure to increased respirable particulate matter, which is consistent with the development of inflammation in the healthy *Control* group from Day 0 to Day 14.

Antigen exposure results in a mild but significant neutrophilic influx into the airway lumen of both people and horses within 5 hours, which spontaneously resolves within days/hours in healthy subjects despite continued exposure to the stimulus.^14^, ^27^, ^28^ We therefore elected to introduce horses to the stabled environment 7 days before initial testing, to enable habituation to the environment and allow any transient neutrophilia induced by transport, or antigen exposure in the new stable environment to resolve in healthy horses. While we considered 7 days sufficient for horses to normalize to this environment, prolonged exposure elicited an immune response in the *Control* group. Stabling is a risk factor for the development of airway inflammation,^29^ with healthy control horses developing profound airway neutrophilia (27.6%) after 3 weeks.^12^ Consistent with previous reports,^12^, ^29^ 3 of the 4 control horses developed airway inflammation, with up regulation of TNF‐α potentially driving neutrophil recruitment into the airways. In healthy horses, TNF‐α, IL‐10, and IL‐12 were up‐regulated, and IL‐4, IL‐5, IL‐23, IL‐1β, and IFN‐γ were down‐regulated in response to prolonged exposure to a dusty, stabled environment. IL‐4 and IL‐5 are central T2 cytokines (Figure 1). Conversely, IL‐12 is secreted by T lymphocytes, and plays as a key role in the differentiation of T0 into T1 cells. We acknowledge that a limitation of this study is the small size of the *Control* group, which has the potential to result in a type II error, and additional cytokine changes might have been identified had there been greater power. In this study, the response of healthy horses to sustained exposure to a sub‐optimal environment was the development of cytological airway inflammation in 3 out of 4 horses, accompanied by a shift away from a T2 cytokine response, toward a T1 cytokine response. This is likely a healthy, normal response to antigenic stimuli; the development of airway inflammation and shift toward a T1 response in *Control* horses needs to be considered when assessing changes in inflammatory gene expression in the *Dex* and *Saline* treatment groups. To address this, our analysis used a relative differential expression between the control group horses and the other 2 treatment groups.

Treatment with nebulized administration of saline was not associated with any improvement in inflammatory cytology, however, nor was there any significant difference in the relative expression of any gene investigated over time (Day 14 vs Day 0). Given that there was a shift toward a T1 response in *Control* horses, this could be interpreted as a beneficial effect associated with saline nebulization. Furthermore, saline treatment was associated with down‐regulation of multiple inflammatory cytokines, in comparison to both asthmatic horses treated with nebulized administration of dexamethasone, as well as *Control* horses. Interestingly, nebulized administration of saline was also associated with a significant decrease in *Streptococcus* in horses with asthma.^25^ However, any potential beneficial effects associated with nebulized administration of saline were not able to counteract the overwhelming effect of prolonged exposure to a dusty environment. It is possible that nebulizing 3 mL of 0.9% saline is not effective at treating airway inflammation, which is why it is was used as a placebo. Interestingly, a pilot study which initially used nebulized administration of saline as a control was redesigned after 2 horses assigned to the initial saline treatment had severe exacerbation of asthma.^30^ However, concentration (0.9% or 3% saline) and dose volume were not reported.^30^ Further studies investigating the use of nebulized saline in the treatment of asthma in horses are warranted.

## 5 CONCLUSIONS

Nebulization of 5 mg of dexamethasone sodium phosphate does not induce lung inflammation in healthy horses,^1^ however, this treatment also fails to demonstrate improvement in lung mechanics in horses with severe asthma,^2^, ^3^ justifying the administration of 15 mg in the present study. However, nebulization of 15 mg dexamethasone sodium phosphate was associated with a mixed inflammatory immune response. Treatment of asthmatic horses with nebulized saline reduced expression of inflammatory cytokine mRNA, although was not associated with an improvement of cytologic inflammation. Improving air quality is an important component of treatment of asthma and should be emphasized when creating treatment plans for the management and prevention of clinical disease.

## CONFLICT OF INTEREST DECLARATION

Authors declare no conflict of interest.

## OFF‐LABEL ANTIMICROBIAL DECLARATION

Authors declare no off‐label use of antimicrobials.

## INSTITUTIONAL ANIMAL CARE AND USE COMMITTEE (IACUC) OR OTHER APPROVAL DECLARATION

This study was conducted in strict accordance with the recommendations of the Canadian Council of Animal Care. The University of Calgary Veterinary Animal Care Committee approved the study (#AC17‐0097) and written consent was obtained from the horses' agent.

## HUMAN ETHICS APPROVAL DECLARATION

Authors declare human ethics approval was not needed for this study.
